# IsoMIF Finder: online detection of binding site molecular interaction field similarities

**DOI:** 10.1093/bioinformatics/btv616

**Published:** 2015-10-25

**Authors:** Matthieu Chartier, Etienne Adriansen, Rafael Najmanovich

**Affiliations:** ^1^Department of Biochemistry, Faculty of Medicine and Health Sciences, University of Sherbrooke, Sherbrooke, J1H 5N4 QC, Canada and; ^2^Telecommunication and computer network engineering, Télécom Lille, Lille 1 University, 59650 Villeneuve-d'Ascq, France

## Abstract

**Summary:** IsoMIF Finder is an online server for the identification of molecular interaction field (MIF) similarities. User defined binding site MIFs can be compared to datasets of pre-calculated MIFs or against a user-defined list of PDB entries. The interface can be used for the prediction of function, identification of potential cross-reactivity or polypharmacological targets and drug repurposing. Detected similarities can be viewed in a browser or within a PyMOL session.

**Availability and Implementation:** IsoMIF Finder uses JSMOL (no java plugin required), is cross-browser and freely available at bcb.med.usherbrooke.ca/imfi.

**Contact:** Rafael.Najmanovich@Usherbrooke.ca

**Supplementary information:** Supplementary data are available at *Bioinformatics* online.

## 1 Background

Binding site similarities capture local physico-chemical correspondences between two protein structures, in particular cavities, based on functional pseudocenters (Shulman-Peleg *et al.*, 2005), all surface atoms (Najmanovich *et al.*, 2008), electrostatic surfaces (Kinoshita *et al.*, 2007) or molecular interaction fields (MIFs) (Chartier and Najmanovich, 2015) among others. Applications include, the prediction of molecular function, detection of cross-reactivity (Weber *et al.*, 2004; Xie *et al.*, 2009) and polypharmacological targets (Kalliokoski and Vulpetti, 2011), druggability analyses (Campagna-Slater *et al.*, 2011), prediction of fragments or bioisosteric replacements (Tang and Altman, 2014; Wood *et al.*, 2012), drug repurposing and classification (Allali-Hassani *et al.*, 2007; Najmanovich *et al.*, 2007), etc. We present IsoMIF Finder, a versatile web-server for the detection of MIF similarities with IsoMIF (Chartier and Najmanovich, 2015). IsoMIF uses 6 probes (hydrophobic, aromatic, H-bond donor/acceptor and positive/negative charge) and a coarse-grained force field to define MIFs measured in cavities and a graph matching procedure to detect MIF similarities. Web-servers exist for some other methods e.g. SuMo (Jambon *et al.*, 2005), pdbFun (Ausiello *et al.*, 2005), eF-seek (Kinoshita *et al.*, 2007), SiteEngine (Shulman-Peleg *et al.*, 2005), ProBiS server (Konc and Janežič, 2014) and IsoCleft Finder (Kurbatova *et al.*, 2013).

## 2 Submitting a job

The query MIFs are calculated in cavities of a PDB structure. The user first provides a PDB (Berman *et al.*, 2007) structure by entering a 4 letter-code or uploads its own. The user then chooses to find the top N largest cavities in the PDB or to find the cavity where a specified ligand is bound. Cavities are identified with the Get_Cleft algorithm (Gaudreault *et al.*, 2015), our in-house implementation of Surfnet (Laskowski, 1995). The user then selects the comparison set, either one of four pre-calculated MIF datasets or a user defined PDB structure list. The four datasets are the human purinome with 2643 MIFs calculated around purine containing ligands bound to human proteins, the scPDB with 8077 MIFs calculated in ligand binding sites (Desaphy *et al.*, 2015), a non-redundant PDB pisces dataset (Wang and Dunbrack, 2003) with 14 082 entries and a dataset of 412 entries of structures of drugs bound to their primary targets (or homologue) taken from the PDB website. As mentioned, the user can alternatively provide a list of PDB structures and for each specify the top N largest cavities to find or one with a specified bound ligand. The MIFs of the comparison set will be calculated in these cavities. Finally, several parameters can be defined and optimal values for each are chosen by default. We refer the readers to the original IsoMIF publication for an explanation of these parameters and other methodological details (Chartier and Najmanovich, 2015) and to the online help guide.

## 3 Cropping the cavities

The cavities identified can sometimes be very large and include non-druggable cavities. By cropping the cavities, the user ensures the query MIFs are calculated in regions of interest. For this, a 3D molecule viewer for each query cavity (Supplementary Fig. S1) shows cavity lining residues that can be unchecked, cropping the cavity volume within 3 Å radius of their atoms. If the user provided a list of PDB entries for the comparison set, a table lists the cavities found with corresponding information like chains in contact, bound molecules and number of residues in contact (Supplementary Fig. S2). Each cavity can be visualized within the protein structure (GIF and PNG) and the user can select only the cavities of interest for the comparison.

## 4 Results page

A results table for each query cavity lists the top hits found (Supplementary Fig. S3) and can be downloaded in CSV format. The top hits can be sorted by Tanimoto similarity or number of nodes (similar probes detected). Additional data provided includes *Z*-score, the search space (number of grid points sampled), the crystalized ligand around which the hit MIF is calculated (for purinome, scPDB and drug-target datasets only), the PFAM family, Uniprot and protein name. A snapshot of the detected similarities can be visualized in the browser with a GIF or PNG image. Downloadable PyMOL sessions show the query (green) and target hit (cyan) structures with bound molecules and MIF similarities, all superimposed using the transformation matrix that optimally minimizes the pairwise distance between matched probes. The similar probes are shown using spheres coloured by interaction type: hydrophobic (cyan), aromatic (orange), H-bond donor (blue), acceptor (red), positive (green) and negative (magenta) charge. Bigger spheres represent probes from the query protein and smaller spheres from the comparison set protein. Semi-transparent spheres represent the initial MIFs of each protein. The matched probes and the initial MIFs for each probe have individual selections in PyMOL making it easy to analyse the different objects individually (Supplementary Fig. S4).

## 5 Example application

**Fig. 1.**
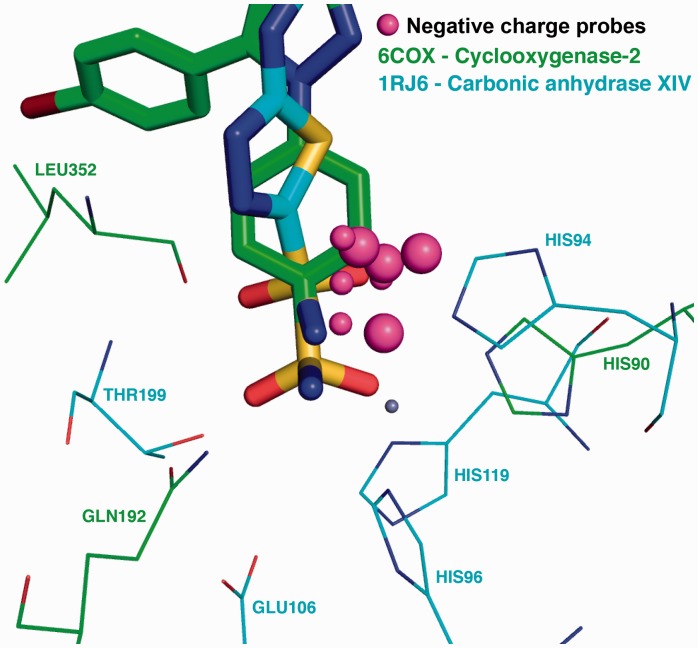
Negatively charged probe similarities between celecoxib binding site of PDB 6COX and acetazolamide binding site of 1RJ6

High level of binding site MIF similarity suggests the cavities might share similar molecular functions. In other words, a drug bound in a one could bind the other. Experimental evidence suggests that carbonic anhydrases can be cross-reactivity targets of celecoxib, a COX-2 specific inhibitor and that this approved drug could be repurposed to treat glaucoma (Weber *et al.*, 2004). When using the celecoxib binding site of COX-2 (PDB 6COX) as query, carbonic anhydrase XIV (PDB 1RJ6) is found within the top hits of the drug-target dataset (Fig. 1; created using the PyMOL session of the MIF similarities). Both ligands have a sulfonamide group surrounded by histidines in both proteins causing the negative charge similarities (magenta spheres). In 6COX, HIS90 could interact with the sulfonamide via hydrogen bonding while in 1RJ6 the interaction would be mediated by a Zn^2+^ atom (small grey sphere) coordinated by HIS94 119 and 96. Hydrogen bond donor and acceptor similarities were also found near the sulfonamide (not shown for clarity). The hydroxyl of THR199 in 1RJ6 creates a region favorable for H-bond acceptor probes, similarly to the amine of GLN192 in 6COX. The same detailed analysis could be done for other probes. This example highlights how the results of IsoMIF Finder can be used to analyse binding site similarities, the underlying structural determinants in a context of drug repurposing and analysis of cross-reactivity.

## Supplementary Material

Supplementary Data
