# Supplementary material for: IsoMIF Finder: online detection of binding site molecular interaction field similarities
Source: Bioinformatics. 2015 Oct 25;32(4):621–3. doi: 10.1093/bioinformatics/btv616 (PMC4743630; doi:10.1093/bioinformatics/btv616)
Supplement: Supplementary Data [file supp_32_4_621__index.html]

IsoMIF Finder: online detection of binding site molecular interaction field similarities — Supplementary Data 

# IsoMIF Finder: online detection of binding site molecular interaction field similarities

## Supplementary Data

files

- Supplementary Data - pdf file
