# Supplementary material for: IsoMIF Finder: online detection of binding site molecular interaction field similarities
Source: Bioinformatics. 2015 Oct 25;32(4):621–3. doi: 10.1093/bioinformatics/btv616 (PMC4743630; doi:10.1093/bioinformatics/btv616)
Supplement: Supplementary Data [file supp_btv616_IsoMIF_Finder_Supplementary_150924.pdf]

IsoMIF Finder: Online Detection of Binding Site Molecular Interaction Field Similarities

Matthieu Chartier[1], Etienne Adriansen[2] and Rafael Najmanovich[1,*]

[1] Department of Biochemistry, Faculty of Medicine and Health Sciences, University of Sherbrooke, 12e Avenue Nord, Sherbrooke, J1H 5N4, Québec, Canada, [2] Telecommunication and computer network engineering, Télécom Lille, Lille 1 University, Rue Guglielmo Marconi, 59650 Villeneuve-d'Ascq, France

*To whom correspondence should be addressed.

Associate Editor: XXXXXXX

Received on XXXXX; revised on XXXXX; accepted on XXXXX

Abstract

Summary: IsoMIF Finder is an online server for the identification of molecular interaction field (MIF) similarities. User defined binding site MIFs can be compared to datasets of pre-calculated MIFs or against a user-defined list of PDB entries. The interface can be used for the prediction of function, identification of potential cross-reactivity or polypharmacological targets and drug repurposing. Detected similarities can be viewed in a browser or within a PyMOL session.

Availability and Implementation: IsoMIF Finder uses JSMOL (no java plugin required), is cross-browser and freely available at bcb.med.usherbrooke.ca/imfi.

Contact: Rafael.Najmanovich@Usherbrooke.ca

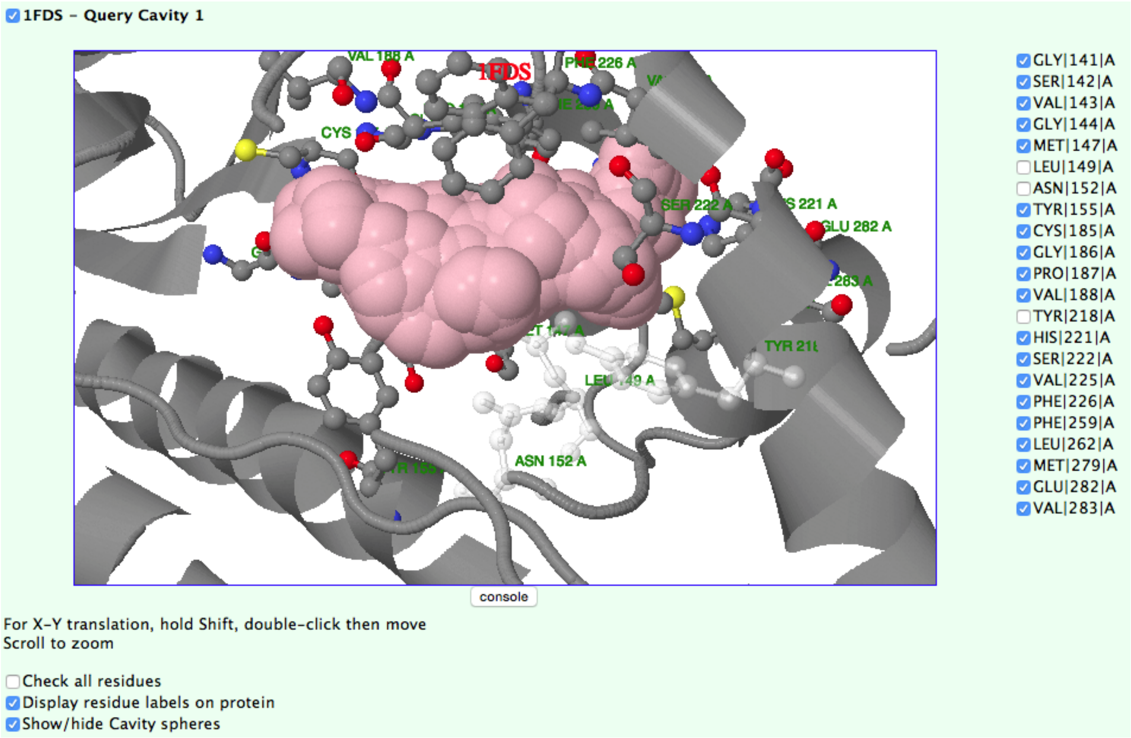

Figure S1. The molecule viewer shows the initial volume of the cavity (light red spheres) and the ligand (if defined) is in black sticks. Cavity lining residues are listed with checkboxes and shown in sticks in the viewer. Three residues are unchecked from the list above, shown with semi-transparent sticks in the viewer. This will crop the volume within 3Å of any atoms of the unchecked residues. On top left, next to the cavity name, the user can uncheck this query cavity to avoid the comparison of its MIF to the comparison set. All residues can be checked at once, the residue labels can be toggled on or off on the structure and the cavity volume sphere's visibility can be toggled on and off.

A

2. Select cavities from the custom PDB list

☑	PDB	Cavity rank	HETATMs specified [Res. Num. Chain Alt.]	Chains in Contact	HETATM(s) within the cavity	Nb. residues in contact	Nb. cavity spheres	GIF	PNG
☑	1E3R	1	–	B	AND 801 B	28	282	GIF	PNG
☑	1E3R	2	–	A	AND 801 A	23	204	GIF	PNG
☑	1E3R	3	–	B	–	8	93	GIF	PNG
☑	1E3R	4	–	A	–	9	74	GIF	PNG
☑	1E3R	5	–	A, B	–	9	74	GIF	PNG
☑	1J99	1	AND 401 A A	A	AND 401 A A<br>IOD 300 A<br>AND 401 A B	55	1141	GIF	PNG
☑	1LHU	2	EST 301 A –	A	EST 301 A	22	296	GIF	PNG
☑	1LHW	1	–	A	ESM 301 A	31	301	GIF	PNG
☑	3TEM	2	FAD 232 A –	A, B	FAD 232 A<br>6A1 501 B	45	1998	GIF	PNG

B

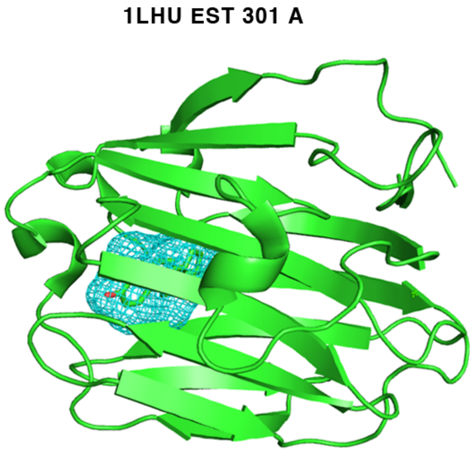

Figure S2. (A) If a user entered a custom PDB list for the comparison set, the cavities found are listed in a table. Several information is given allowing the user to select only cavities of interest. (B) For each cavity, a GIF and PNG file allow the user to inspect the volume of the cavities (blue mesh) where the MIF will be calculated. The cavity of PDB entry 1LHU (shown in green cartoon) was entered by the user in the comparison set with a ligand (EST 301 A).

Cavity 1
View MIF: PyMOL | GIF | PNG
CSV file sorted by: tanimoto | nodes

Sort by: Tanimoto | Nodes
Different Pfam: Yes | No

#	Protein	Tanimoto	Zscore	Nodes	Zscore	Search Space 6COX	Search Space target	Crystalized Ligand	Pfam	Uniprot	Pro[illegible]	[illegible]	[illegible]	[illegible]
1	4COX_IMN701B–	0.4635	3.57	74	2.10	87	99	IMN–701–B––	PF03098	Q05769	CY[illegible]	[illegible]	[illegible]	[illegible]
2	1TD7_NFL2001A–	0.3626	2.08	54	0.63	87	69	NFL–2001–A––	PF00068	P60045	PH[illegible] ISO[illegible]	[illegible]	[illegible]	[illegible]
3	2Q7I_TES205A–	0.3552	1.97	58	0.92	87	78	TES–205–A––	PF00104	P10275	AN[illegible]	[illegible]	[illegible]	[illegible]
4	1ZZ1_SHH2452A–	0.3547	1.97	57	0.85	87	77	SHH–2452–A––	PF00850	Q70I53	HIS[illegible] DE[illegible] AM[illegible]	[illegible]	[illegible]	[illegible]
5	1RJ6_AZM400A–	0.3448	1.82	47	0.11	87	58	AZM–400–A––	PF00194	Q9WVT6	CARBONIC ANHYDRASE XIV	pml	GIF	PNG
6	2WX2_TPF1460B–	0.3382	1.72	69	1.73	87	112	TPF–1460–B––	PF00067	Q7Z1V1	LANOSTEROL 14–ALPHA–DEMETHYLASE	pml	GIF	PNG
7	3RFM_CFF330A–	0.3280	1.57	59	0.99	87	82	CFF–330–A––	PF00001	P29274	ADENOSINE RECEPTOR A2A	pml	GIF	PNG
8	4U5J_RXT601B–	0.3263	1.55	60	1.07	87	91	RXT–601–B––	PF07714	P00523	PROTO–ONCOGENE TYROSINE–PROTEIN KINASE SRC	pml	GIF	PNG
9	3TVX_PNX902B–	0.3249	1.53	61	1.14	87	87	PNX–902–B––	PF00233	P27815	CAMP–SPECIFIC 3' –5'–CYCLIC PHOSPHODIESTERASE 4A	pml	GIF	PNG

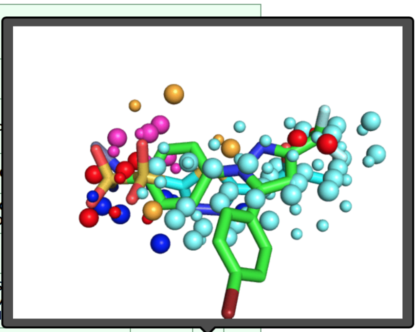

Figure S3. Results page for query 6COX with MIF calculated around ligand S58 701 A. The similarity for the top hits can be visualized by hovering the mouse over the GIF or PNG image as shown for top hit 1RJ6 and the PyMOL sessions can be downloaded individually for the top hits.

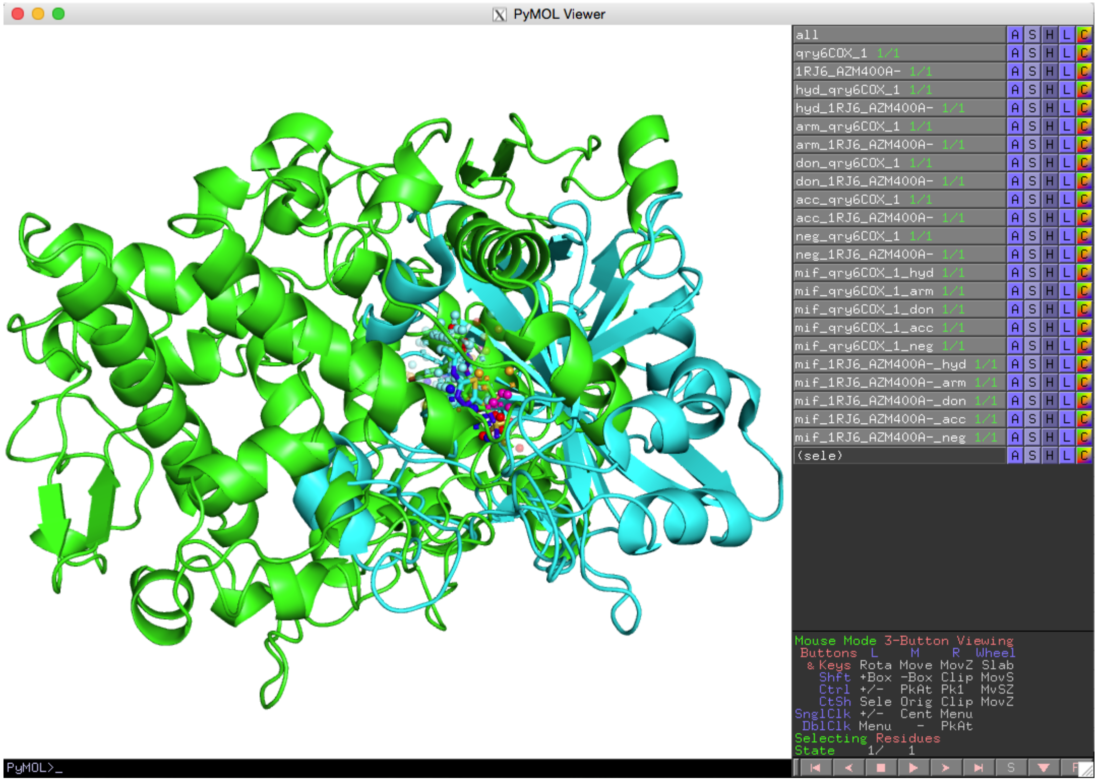

Figure S4. The PyMOL session above shows the similarities between query 6COX and 1RJ6. It contains different objects : *qry6COX_1* is the query protein structure, *1RJ6_AZM400A-* is the top hit structure. The objects that start with *hyd_*, *arm_*, *don_*, *acc_*, *neg_* and *pos_* are the probes found similar in both proteins. The objects that start with *mif_* represent all the probes (shown in semi-transparent) with favorable interaction energy found in each cavity.
